# A Visit to a Swiss Hospital

**Published:** 1909-05-01

**Authors:** Conrad W. Thies

**Affiliations:** Secretary to the Royal Free Hospital, Gray's Inn Road, London


### A VISIT TO A SWISS HOSPITAL.

BY CONRAD W. THIES, Secretary to the Royal Free Hospital, Gray's Inn Road, London.

The Insel Hospital at Berne is beautifully situated on the outskirts of the city, on rising ground sloping to the south-east and commanding magnificent views of the snow-capped mountains of the Bernese Oberland. It is built on the pavilion system, the various buildings being connected by uncovered asphalted paths, which are interspersed with lawns, flower beds, and shrubberies. The whole of the buildings are heated and ventilated by an elaborate hot-air system, which is somewhat allied to what is known in this country as the "plenum system." The air, after being filtered, is conveyed from the boiler-house through underground conduits and pipes to the various parts of the institution. The vitiated air is sucked out by means of fans and carried back to the flue of the boiler-house chimney.

There is nothing very special to remark respecting the general equipment of the wards and various departments, which are mainly on the lines of the modern German hospitals. The provision made for skin diseases is very complete, for such complaints are somewhat common in the canton of Berne. The hospital is a combination of two old institutions—namely, the Insel (or Island) Hospital, with 260 beds, and the "Outer Hospital" for incurable cases, with 120 beds, of which twenty are reserved for children. These two institutions now form one Corporation, which is recognised by the State and, under the general inspection of the Government, administers their affairs and is empowered to acquire property.

Both the hospitals are available for the purposes of medical education, and it is specially provided in the regulations that "They are to keep pace with the ever-growing needs of science." Applications for admission must be made upon a special form, which is provided by the hospital. This form demands much detailed information as to the means and circumstances of the applicant for treatment, and it must be verified by the Mayor or Magistrate of the district where the patient lives.

In all cases the poor citizens of Berne have the first claim for admission, then the poorest of the applicants from the neighbouring villages. The Directors decide in each case whether the patient shall be admitted free or what payment shall be paid. In many instances the payment is made by the Canton, when the patient is too poor to make any payment. The payments demanded vary according to the means of the patients, and range from 1 to 15 francs per day. The rest of the cost of maintaining the hospital is provided by subsidies from the State and Commune, and by the benefactions of private individuals.

During the middle ages pious bequests for charitable purposes were very common in Switzerland; these bequests were, however, mostly devoted to religious purposes, such as the building and endowment of churches and convents, also for the maintenance of masses for the dead and special services. The "Insel Hospital" owes its foundation to the benefactions of Anna Seiler, who lived in the fourteenth century. The Mayor and Council of Berne accepted the bequest of Anna Seiler and loyally carried out her wishes. They executed a reciprocal deed of trust and thereby declared that they would take charge and look after the hospital and adhere to the instructions and conditions made by the foundress. From that day to the present the authorities of Berne have continued to superintend the hospital, which is still one of the best-equipped and managed hospitals in Switzerland.

### A Quaint Will.

The following is a literal translation of the wording of the original Deed of Trust of the above Hospital:—

"In the name of God Amen, I Anna Seiler, Citizeness of and dwelling in Berne, now in good health and of clear mind and with the advice and permission of the Mayor and of the Council and of the Two Hundred of Berne, for the benefit of my soul and of the souls of all my ancestors and of all believers, for the curing and comforting continually and for ever of the Town and Citizens of Berne and to perform the works of mercy, viz.: (1) to feed the hungry, (2) to give drink to the thirsty, (3) to shelter the stranger, (4) to clothe the naked, (5) to tend the sick, (6) to comfort the prisoners, (7) to bury the dead. In order that this may be accomplished have arranged founded and made by the hand of Nicholas of Muleron Citizen of Berne, my Executor, a perpetual Hospital, wherein shall be for ever 13 Bedridden and indigent persons and 3 other reputable persons, who are also themselves needy to nurse and serve the bedridden ones, and if the needy ones or the persons at any time go away so shall always be chosen another needy and bedridden or a person of this town in the place of those gone away.

"I also appoint order and make with these deeds and with the hand of the aforesaid Executor that after my death the Mayor the Council and the 200 of Berne and their successors and no one else shall establish the aforesaid Hospital and provide same with Governors and with other things whatever is necessary for it as they then think."

